# Efficient Use of a Crude Drug/Herb Library Reveals Ephedra Herb As a Specific Antagonist for T_H_2-Specific Chemokine Receptors CCR3, CCR4, and CCR8

**DOI:** 10.3389/fcell.2016.00054

**Published:** 2016-06-07

**Authors:** Kazuhiko Matsuo, Keiichi Koizumi, Mitsugu Fujita, Toshio Morikawa, Michiko Jo, Naotoshi Shibahara, Ikuo Saiki, Osamu Yoshie, Takashi Nakayama

**Affiliations:** ^1^Division of Chemotherapy, Faculty of Pharmacy, Kindai UniversityHigashiōsaka, Japan; ^2^Division of Kampo Diagnostics, Institute of Natural Medicine, University of ToyamaToyama, Japan; ^3^Department of Microbiology, Faculty of Medicine, Kindai UniversityŌsakasayama, Japan; ^4^Department of Pharmaceutical Food Sciences, Pharmaceutical Research and Technology Institute, Kindai UniversityHigashiōsaka, Japan; ^5^Division of Pathogenic Biochemistry, Institute of Natural Medicine, University of ToyamaToyama, Japan

**Keywords:** chemokine receptor, CCR3, CCR4, antagonist, Ephedra Herb, maoto

## Abstract

Chemokine receptors CCR3 and CCR4 are preferentially expressed by T_H_2 cells, mast cells, and/or eosinophils, all of which are involved in the pathogenesis of allergic diseases. Therefore, CCR3 and CCR4 have long been highlighted as potent therapeutic targets for allergic diseases. Japanese traditional herbal medicine Kampo consists of multiple crude drugs/herbs, which further consist of numerous chemical substances. Recent studies have demonstrated that such chemical substances appear to promising sources in the development of novel therapeutic agents. Based on these findings, we hypothesize that Kampo-related crude drugs/herbs would contain chemical substances that inhibit the cell migration mediated by CCR3 and/or CCR4. To test this hypothesis, we screened 80 crude drugs/herbs to identify candidate substances using chemotaxis assay. Among those tested, Ephedra Herb inhibited the chemotaxis mediated by both CCR3 and CCR4, Cornus Fruit inhibited that mediated by CCR3, and Rhubarb inhibited that mediated by CCR4. Furthermore, Ephedra Herb specifically inhibited the chemotaxis mediated by not only CCR3 and CCR4 but CCR8, all of which are selectively expressed by T_H_2 cells. This result led us to speculate that ephedrine, a major component of Ephedra Herb, would play a central role in the inhibitory effects on the chemotaxis mediated by CCR3, CCR4, and CCR8. However, ephedrine exhibited little effects on the chemotaxis. Therefore, we fractionated Ephedra Herb into four subfractions and examined the inhibitory effects of each subfraction. As the results, ethyl acetate-insoluble fraction exhibited the inhibitory effects on chemotaxis and calcium mobilization mediated by CCR3 and CCR4 most significantly. In contrast, chloroform-soluble fraction exhibited a weak inhibitory effect on the chemotaxis mediated by CCR8. Furthermore, maoto, one of the Kampo formulations containing Ephedra Herb, exhibited the inhibitory effects on the chemotaxis mediated by CCR3, CCR4, and CCR8. Taken together, our data suggest that these crude drugs/herbs might be useful sources to develop new drugs targeting T_H_2-mediated allergic diseases.

## Introduction

Chemokines are a structurally related set of proteins which, in coordination, recruit various leukocytes into target sites via corresponding receptors. In human, there are at least 44 chemokines and 18 signal transducing receptors (Zlotnik and Yoshie, 2000, 2012; Yoshie et al., 2001). Chemokines play important roles in various biological processes such as homeostatic migration and the homing of lymphocytes, inflammatory mobilization of leukocytes, cell migration, and homing during development, angiogenesis, and cancer metastasis (Fujita et al., 2015). Chemokine receptors belong to the seven-membrane G protein-coupled receptor (GPCR) family (Zlotnik and Yoshie, 2000; Yoshie et al., 2001; Zlotnik and Yoshie, 2012). As a number of drugs targeting GPCRs have been successfully developed thus far (Wood and Armour, 2005; Subramaniam et al., 2012; Martin-Blondel et al., 2016), chemokine-chemokine receptor axes are considered to be promising drug targets for inflammatory and immunological diseases.

Allergic inflammation is a critical feature of several allergic diseases such as atopic dermatitis, allergic rhinitis, and asthma (Ng and Wang, 2015; Pols et al., 2016; Weidinger and Novak, 2016). In particular, T_H_2 cells, mast cells, and eosinophils are involved in the pathogenesis of these allergic diseases as major effector cells (Wynn, 2015). In this regard, T_H_2 cell-derived cytokines such as IL-4, IL-5, and IL-13 stimulate IgE production by B cells, and induce migration, activation, and growth of mast cells and eosinophils (Del Prete, 1998). Thus, the selective migration of these effector cells results in allergic inflammation. Therefore, a potent strategy to overcome the allergic diseases would be to interfere with the migration of such effector cells. In this regard, CC chemokine receptor 3 (CCR3) is selectively expressed by eosinophils, basophils, and a part of T_H_2 cells that play major roles in allergic diseases (Daugherty et al., 1996; Kitaura et al., 1996; Sallusto et al., 1997; Uguccioni et al., 1997). The following CC chemokines function as ligands for CCR3: eotaxin/CCL11, eotaxin-2/CCL24, eotaxin-3/CCL26, RANTES/CCL5, MCP-2/CCL8, MCP-3/CCL7, and MCP-4/CCL13 (Daugherty et al., 1996; Kitaura et al., 1996, 1999; Ponath et al., 1996; Forssmann et al., 1997; Heath et al., 1997). Among them, the three eotaxins (CCL11, CCL24, and CCL26) exhibit the highest specificity for CCR3 (Yoshie et al., 2001; Zlotnik and Yoshie, 2012). In addition, their expression levels frequently increase in allergic inflammatory sites (Blanchard et al., 2006). The chemokine CCR4 is dominantly expressed by T_H_2 cells (Yamamoto et al., 2000), and its ligands are CCL17 and CCL22 (Imai et al., 1997, 1998). The importance of the CCR4 axis in allergic diseases has been demonstrated in mouse models of atopic dermatitis and asthma (Gonzalo et al., 1999; Vestergaard et al., 1999; Kawasaki et al., 2001). Furthermore, we and others have demonstrated that the serum levels of CCL17 and CCL22 substantially increase in patients with allergic diseases (Kakinuma et al., 2001, 2002; Fujisawa et al., 2002; Horikawa et al., 2002) and that the serum levels of CCL17 correlate particularly with the disease activities of atopic dermatitis (Kataoka, 2014). Based on these data, the Japanese Pharmaceutical and Medical Devices Agency has approved a serum CCL17 ELISA kit (Alaport® TARC) as a clinical examination tool for atopic dermatitis. Collectively, these data strongly suggest that CCR3 and CCR4 are important therapeutic targets for a variety of allergic diseases.

Japanese traditional herbal medicine Kampo, which has a long-lasting history, consists of multiple crude drugs/herbs, which further consist of numerous chemical substances (Hijikata, 2006). Over 140 Kampo formulations have been approved as ethical drugs in Japanese Pharmacopeia and used clinically for a variety of diseases. Some Kampo formulations are effective in the treatment of inflammatory diseases (Shimizu, 2013). In particular, maoto contains Ephedra Herb, and its major component of Ephedra Herb is an alkaloid ephedrine (The Ministry of Health, Labour and Welfare, 2011). Ephedrine is known to bronchodilating activities and anti-inflammatory effects (The Ministry of Health, Labour and Welfare, 2011). Taken together, these observations suggest that the chemical substances in Kampo-related crude drugs/herbs may be promising sources in the development of novel therapeutic agents.

Based on these findings, we hypothesize that Kampo-related crude drugs/herbs would contain chemical substances that inhibit the cell migration mediated by CCR3 and/or CCR4. To test this hypothesis, we screened 80 crude drugs/herbs to identify candidate substances. Among those tested, three extracts (Cornus Fruit, Rhubarb, and Ephedra Herb) inhibited the cell migration mediated by CCR3 and CCR4. Furthermore, Ephedra Herb specifically inhibited the cell migration mediated by not only CCR3 and CCR4 but CCR8, all of which are selectively expressed by T_H_2 cells. Consistently, maoto, one of the Kampo formulations containing Ephedra Herb, showed potency to inhibit the cell migration mediated by CCR3, CCR4, and CCR8. Taken together, our data suggest that these crude drugs/herbs might be useful sources to develop new drugs targeting T_H_2-mediated allergic diseases.

## Materials and methods

### Crude drugs/herbs and reagents

Crude drugs/herbs were dissolved in H_2_O at a concentration of 10 mg/ml, and the stock solutions were stored at −80°C. The voucher samples of these extracts were reserved in the Cooperative Research Project of Institute of Natural Medicine at University of Toyama. Ephedrine was purchased from Nichi-Iko (Toyama, Japan) Recombinant human chemokines were purchased from R&D Systems (Minneapolis, MN).

### Cells

A mouse pre-B cell line L1.2 was kindly given by Dr. E. Butcher (Stanford University School of Medicine, Stanford, CA). The panels of L1.2 cell lines that stably express human chemokine receptors were generated using a retroviral vector pMX-IRES-EGFP as described previously (Yoshida et al., 1999).

### Chemotaxis assay

The procedure has been described previously (Nakayama et al., 2004). Briefly, chemotaxis assays were performed using 96-well chemoTx chamber (Neuroprobe, Gaithersburg, MD). Cells that migrated into the lower wells were lysed with 0.1% Triton X-100 (Wako, Osaka, Japan) and quantified using PicoGreen dsDNA reagent (Thermo Fisher Scientific, Waltham, MA).

### Calcium mobilization assay

The procedure has been described previously (Nakayama et al., 2010). Briefly, cells were loaded with 3 μM fura 2-AM fluorescence dye (Thermo Fisher Scientific). After washing, the cells were placed on a F3000 fluorescence spectrophotometer (Hitachi, Tokyo, Japan) and stimulated with each recombinant human chemokine. Emission fluorescence at 510 nm was measured upon excitation at 340 and 380 nm, and the fluorescence intensity ratio (R340/380) was obtained.

### Extraction

Dried stems and leaves of Ephedra sinica stapf (50 g) were boiled in 500 mL H_2_O for 50 min, and the decoction was filtered. The filtrate was concentrated through depressurization, and the H_2_O extract (5.22 g) was further suspended in ethyl acetate (EtOAc). The EtOAc phase was extracted and evaporated in vacuo to yield the following fractions: 0.8 g of AcOEt-insoluble fraction, 0.2 g of AcOEt-eluted fraction, and H_2_O-eluted fraction. The H_2_O-eluted fraction was titrated with HCl and further partitioned between CH_3_Cl and 25% ammonia solution to yield extracts of 2.8 and 1.1 g, respectively.

### Statistical analyses

The procedure has been described previously (Otsubo et al., 2015; Yasuda et al., 2015). Briefly, Student's *t*-test was performed to analyze differences between two groups; one-way analysis of variance (ANOVA) with Holm's *post-hoc* test was performed for multiple groups. All data were analyzed using R Environment (R Development Core Team, Vienna, Austria) with EZR plugin version (Kanda, 2013). *P* < 0.05 was considered to be statistically significant.

## Results

### Ephedra herb inhibits the chemotaxis mediated by CCR3, CCR4, and CCR8

**Figure 1:**
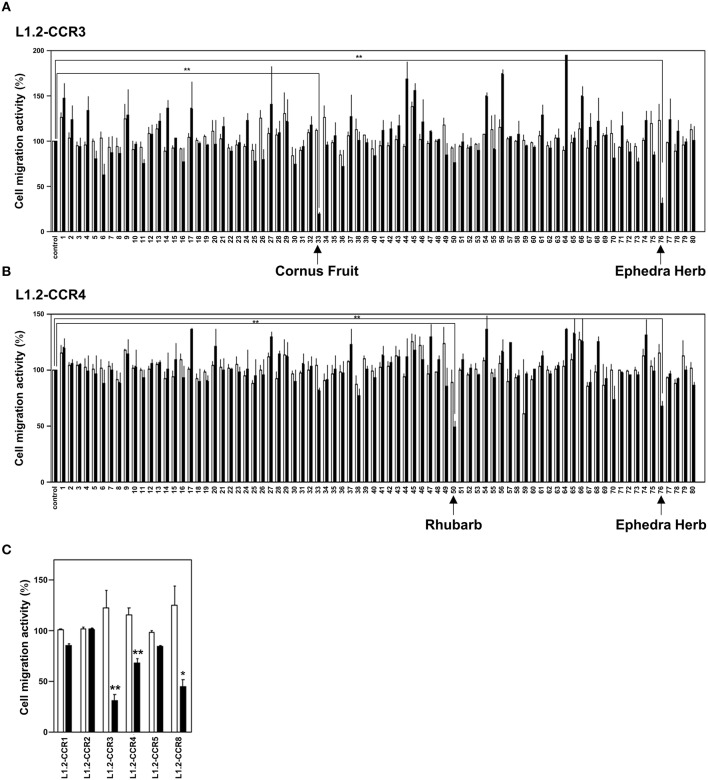
**Ephedra Herb inhibits the chemotaxis mediated by CCR3, CCR4, and CCR8**. **(A)**, Cell migration assay was performed using L1.2 cells stably expressing CCR3 (L1.2-CCR3) and 10 nM CCL11 in the presence of each crude drug/herb extract at 10 μg/ml (open columns) or 100 μg/ml (closed columns). Each experiment was repeated three times. Cell migration activity is shown in a percentage relative to the control (mean ± SE). **(B)**, Cell migration assay was performed using L1.2-CCR4 and 10 nM CCL22 in the presence of each extract at 10 μg/ml (open columns) or 100 μg/ml (closed columns). **(C)**, Cell migration assay was performed using the following cells and corresponding chemokines in the presence of Ephedra Herb at 10 μg/ml (open columns) or 100 μg/ml (closed columns): L1.2-CCR1/CCL5, L1.2-CCR2/CCL2, L1.2-CCR3/CCL11, L1.2-CCR4/CCL22, L1.2-CCR5/CCL5, and L1.2-CCR8/CCL1. Each chemokine was used at 10 nM. *P*-values were based on ANOVA with Holm's *post-hoc* test **(A,B)** and Student's *t*-test **(C)**. ^*^P < 0.05 and ^**^P < 0.01 compared with the controls.

To identify candidates of CCR3 and CCR4 antagonists from a crude drug/herb library, we screened 80 crude drugs/herbs (Table 1) based on chemotaxis assays using L1.2 cell lines that stably express CCR3 (L1.2-CCR3; Figure 1A) and CCR4 (L1.2-CCR4; Figure 1B). As the results, Ephedra Herb inhibited the cell migration of both L1.2-CCR3 and L1.2-CCR4, Cornus Fruit inhibited that of L1.2-CCR3, and Rhubarb inhibited that of L1.2-CCR4 (Figures 1A,B). We confirmed that there were no cytotoxicity at these concentrations using a cell viability assay (data not shown). Among the crude drugs/herbs tested, we decided to focus on Ephedra Herb because it most effectively inhibited the cell migration mediated by both CCR3 and CCR4. Given that CCR3 and CCR4 have structural similarity to CCR1, CCR2, CCR5, and CCR8, we next examined the receptor specificity of Ephedra Herb using L1.2-CCR1, L1.2-CCR2, L1.2-CCR3, L1.2-CCR4, L1.2-CCR5, and L1.2-CCR8 (Figure 1C). As the results, Ephedra Herb specifically inhibited the chemotaxis mediated by CCR8 in addition to CCR3 and CCR4. As T_H_2 cells selectively express CCR3, CCR4, and CCR8, these data suggest that Ephedra Herb has a potency to strongly suppress cell migration of T_H_2 cells and T_H_2 cell-mediated allergic reactions.

**Table 1:** **The list of a crude drug/herb library**.

**No**.	**Crude drugs/herbs**	**No**.	**Crude drugs/herbs**	**No**.	**Crude drugs/herbs**
1	Artemisia Capillaris Flower	31	Saffron	61	Japanese Angelica Root
2	Turmeric	32	Gardenia Fruit	62	Peach Kernel
3	Corydalis Tuber	33	Cornus Fruit	63	Ginseng
4	Astragalus Root	34	Zanthoxyrum Fruit	64	Fritillaria Bulb
5	Scutellaria Root	35	Jujube Seed	65	Ophiopogon Tuber
6	Phellodendron Bark	36	Rehmannia Root	66	Mentha Herb
7	Coptis Rhizome	37	Eleutherococcus senticosus	67	Pinellia Tuber
8	Polygala Root	38	Lycium Bark	68	Angelica Dahurica Root
9	Zedoary	39	Processed Rehmannia Root	69	Atractylodes Rhizome
10	Pueraria Root	40	Peony Root	70	Areca
11	Trichosanthes Root	41	Plantago Seed	71	Poria Sclerotium
12	Processed Ginger	42	Ginger	72	Processed Aconite Root
13	Glycyrrhiza	43	Cimicifuga Rhizome	73	Sinomenium Stem and Rhizome
14	Platycodon Root	44	Magnolia Flower	74	Saposhnikovia Root and Rhizome
15	Chrysanthemum Flower	45	Red Peony Root	75	Moutan Bark
16	Immature Orange	46	Cnidium Rhizome	76	Ephedra Herb
17	Notopterygium	47	Atractylodes Lancea Rhizome	77	Hemp Fruit
18	Apricot Kernel	48	Mulberry Bark	78	Coix Seed
19	Sophora Root	49	Perilla Herb	79	Japanese Gentian
20	Schizonepeta Spike	50	Rhubarb	80	Forsythia Fruit
21	Cinnamon Bark	51	Jujube		
22	Red Ginseng	52	Alisma Rhizome		
23	Cyprus Rhizome	53	Panax Japonicus Rhizome		
24	Magnolia Bark	54	Anemarrhena Rhizome		
25	Achyranthes Root	55	Clove		
26	Euodia Fruit	56	Uncaria Hook		
27	Burdock Fruit	57	Polyporus Sclerotium		
28	Schisandra Fruit	58	Citrus Unshiu Peel		
29	Bupleurum Root	59	Gastrodia Tuber		
30	Asiasarum Root	60	Asparagus Tuber		

### Ethyl acetate (EtOAc)-insoluble fraction of ephedra herb inhibits the chemotaxis mediated by CCR3 and CCR4

**Figure 2:**
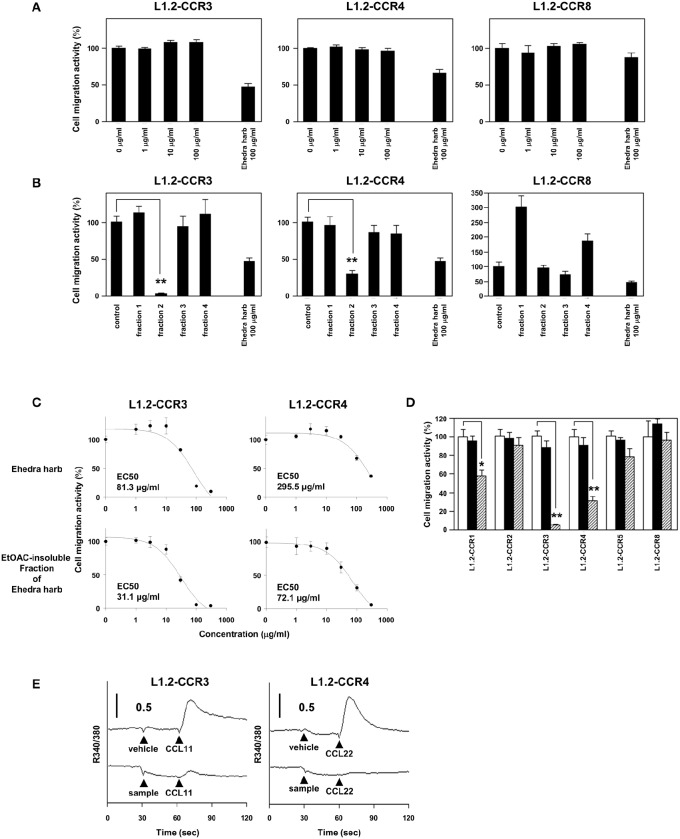
**Ethyl acetate (EtOAc)-insoluble fraction of Ephedra Herb inhibits the chemotaxis mediated by CCR3 and CCR4**. **(A)**, Cell migration assay was performed using the following cells and corresponding chemokines in the presence of ephedrine at the indicated concentrations: L1.2-CCR3/CCL11, L1.2-CCR4/CCL22, and L1.2-CCR8/CCL1. Unfractionated Ephedra Herb was used as a control. Each chemokine was used at 10 nM. Each experiment was repeated three times. Cell migration activity is shown in a percentage relative to the control (mean ± SE). **(B)**, Cell migration assay was performed using L1.2-CCR3/CCL11, L1.2-CCR4/CCL22, and L1.2-CCR8/CCL1 in the presence of each fraction of Ephedra Herb at 100 μg/ml. **(C)**, Cell migration assay was performed as described in the panel **(B)** in the presence of Ephedra Herb and its EtOAC-insoluble fraction at the indicated concentrations. EC_50_ was also calculated. **(D)**, Cell migration assay was performed using the following cells and corresponding chemokines in the presence of EtOAC-insoluble fraction of Ephedra Herb at 0 μg/ml (open column), 10 μg/ml (closed columns), or 100 μg/ml (gray columns): L1.2-CCR1/CCL5, L1.2-CCR2/CCL2, L1.2-CCR3/CCL11, L1.2-CCR4/CCL22, L1.2-CCR5/CCL5, and L1.2-CCR8/CCL1. Each chemokine was used at 10 nM. **(E)**, Calcium mobilization assay was performed using L1.2-CCR3/CCL11 and L1.2-CCR4/CCL22 in the presence of Ephedra Herb and its EtOAC-insoluble fraction. The cells were loaded with fura 2-AM and stimulated with the corresponding chemokines at 10 nM with or without Ephedra Herb and its EtOAC-insoluble fraction at 100 μg/ml. Intracellular calcium mobilization was measured on a fluorescence spectrophotometer. Each experiment was repeated three times; representative results are presented. *P*-values were based on ANOVA with Holm's *post-hoc* test **(B)** and Student's *t*-test **(A,D)**. ^*^P < 0.05 and ^**^P < 0.01 compared with the controls.

Next, we sought to identify constituents that inhibit the chemotaxis mediated by CCR3 and CCR4. As described above, ephedrine is a major component of Ephedra Herb and possesses bronchodilating activities and anti-inflammatory effects. We therefore addressed whether ephedrine could inhibit the cell migration mediated by CCR3, CCR4, and/or CCR8. However, ephedrine exhibited little inhibitory effects on the cell migration of L1.2-CCR3, L1.2-CCR4, and L1.2-CCR8 (Figure 2A). This result led us to seek for other constituents except ephedrine that inhibit the chemotaxis mediated by CCR3, CCR4, and CCR8. To this end, we fractionated Ephedra Herb to the following four subfractions: EtOAc-soluble (fraction 1), EtOAc-insoluble (fraction 2), CH_3_Cl-soluble (fraction 3), and water-eluted (fraction 4) (Figure 2B). The EtOAc-insoluble fraction (fraction 2) exhibited significant inhibitory effects on the chemotaxis of L1.2-CCR3 and L1.2-CCR4 but not on that of L1.2-CCR8. In contrast, the CH_3_Cl-soluble fraction (fraction 3) partially inhibited the chemotaxis of L1.2-CCR8 alone.

As the EtOAc-insoluble fraction exhibited the significant inhibitory effects on CCR3 and CCR4, we then sought to quantify the inhibitory effects of Ephedra Herb and the EtOAc-insoluble fraction. As the results, both Ephedra Herb and the EtOAc-insoluble fraction inhibited the chemotaxis mediated by CCR3 and CCR4 in a dose-dependent manner (Figure 2C). Furthermore, the inhibitory effects of the EtOAc-insoluble fraction (CCR3: EC_50_ = 31.1 μg/ml; CCR4: 72.1 μg/ml) was significantly stronger than that of Ephedra Herb (CCR3: EC_50_ = 81.3 μg/ml; CCR4: 295.5 μg/ml). We next sought to confirm the receptor specificity of the EtOAc-insoluble fraction of Ephedra Herb (Figure 2D). As the results, the EtOAc-insoluble fraction significantly inhibited the chemotaxis of L1.2-CCR3 and L1.2-CCR4 whereas this fraction partially inhibited the chemotaxis of L1.2-CCR1. In addition, the EtOAc-insoluble fraction significantly inhibited the calcium mobilization induced by CCR3 and CCR4 (Figure 2E). Taken together, these data suggest that the EtOAc-insoluble fraction of Ephedra Herb contains the constituents that function as an antagonist against CCR3 and CCR4 at high levels.

### EtOAc-insoluble fraction of ephedra herb inhibits all ligand-induced chemotaxis mediated by CCR3 and CCR4

**Figure 3:**
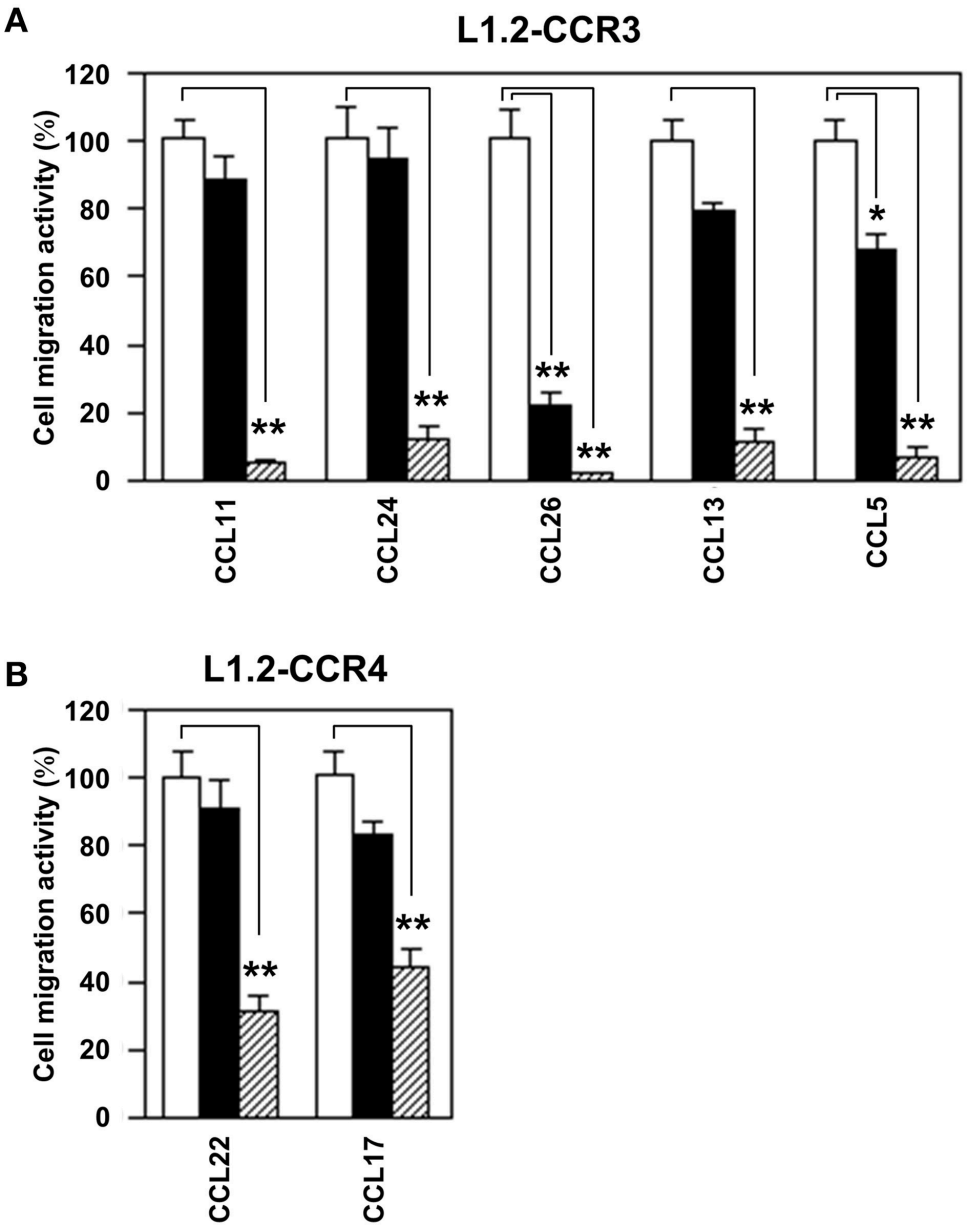
**EtOAc-insoluble fraction of Ephedra Herb inhibits all ligand-induced chemotaxis mediated by CCR3 and CCR4**. **(A)**, Cell migration assay was performed using L1.2-CCR3 and the following chemokines in the presence of the EtOAC-insoluble fraction of Ephedra Herb at 0 μg/ml (open column), 10 μg/ml (closed column), or 100 μg/ml (gray column): CCL11, CCL24, CCL26, CCL13, and CCL5. Each chemokine was used at 10 nM**. (B)**, Cell migration assay was performed using L1.2-CCR4, CCL22, and CCL17 in the presence of the EtOAC-insoluble fraction of Ephedra Herb at 0 μg/ml (open columns), 10 μg/ml (closed columns), or 100 μg/ml (gray columns). Both chemokines were used at 10 nM. Each experiment was repeated three times; representative results are presented. Cell migration activity is shown in a percentage relative to the control (mean ± SE). *P*-values were based on Student's *t*-test. ^*^P < 0.05 and ^**^P < 0.01 compared with the controls.

Subsequently, we addressed whether the EtOAc-insoluble fraction would antagonize any specific ligands for CCR3 and CCR4 (Figure 3). To this end, we used CCL11, CCL24, CCL26, CCL13, and CCL5 as CCR3 ligands and CCL17 and CCL22 as CCR4 ligands. As the results, the EtOAc-insoluble fraction inhibited all ligand-induced chemotaxis of L1.2-CCR3 (Figure 3A) and L1.2-CCR4 (Figure 3B). These data suggest that the EtOAc-insoluble fraction possesses no specific antagonizing activities against the ligands for CCR3 and CCR4 but does directly inhibit these receptors.

### Maoto inhibits the chemotaxis mediated by CCR3, CCR4, and CCR8

**Figure 4:**
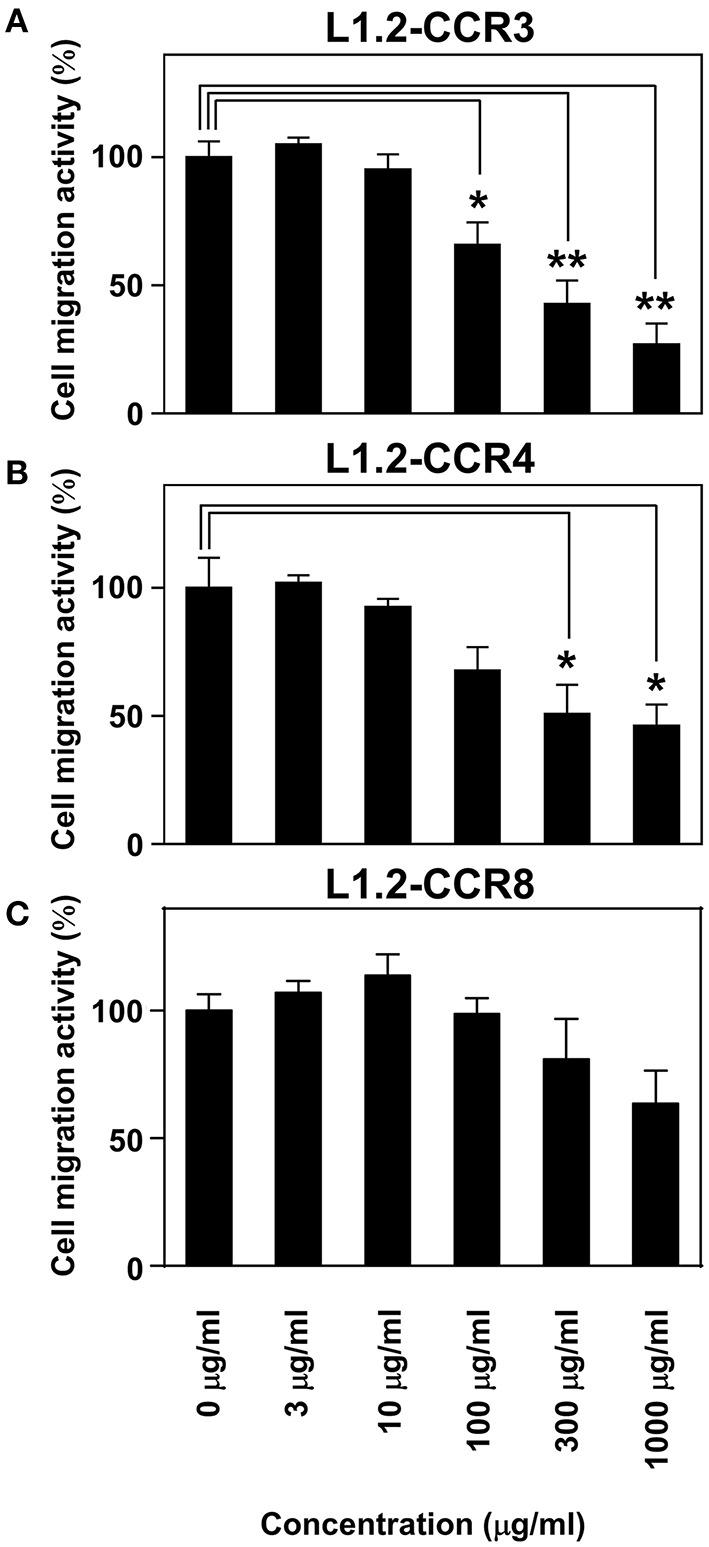
**Maoto inhibits the chemotaxis mediated by CCR3, CCR4, and CCR8**. Cell migration assay was performed using the following cells and corresponding chemokines in the presence of maoto at the indicated concentrations: L1.2-CCR3/CCL11 **(A)**, L1.2-CCR4/CCL22 **(B)**, and L1.2-CCR8/CCL1 **(C)**. Each chemokine was used at 10 nM. Each experiment was repeated three times; representative results are presented. Cell migration activity is shown in a percentage relative to the control (mean ± SE). *P*-values were based on Student's *t*-test. ^*^P < 0.05 and ^**^P < 0.01 compared with the controls.

Maoto is one of the Kampo formulations containing Ephedra Herb and most commonly used in clinical settings. Therefore, we gave importance to examine the inhibitory effects of maoto on the chemotaxis via CCR3, CCR4, as well as CCR8 (Figure 4). As the results, maoto inhibited the chemotaxis of L1.2-CCR3 (Figure 4A), L1.2-CCR4 (Figure 4B), and partially L1.2-CCR8 (Figure 4C) in a dose dependent manner, which is consistent with our observation in Ephedra Herb (Figure 1C). These results suggested that maoto has a potency to inhibit T_H_2 cell migration.

## Discussion

Allergic diseases are caused by a T_H_2-dominant condition, which is characterized by the infiltration of T_h_2 cells, eosinophils, and mast cells. T_H_1 and T_H_2 cells express distinct patterns of chemokine receptors that enable selective migration toward different types of inflammation. T_H_2 cells preferentially express CCR3, CCR4, and CCR8 while Th1 cells preferentially express CCR5 and CXCR3. Furthermore, CCR3 is the major receptor expressed on eosinophils and basophils. Thus, CCR3 and CCR4 have been paid attention as potent therapeutic targets for allergic diseases. In this study, we demonstrated that three crude drugs/herbs to possess the antagonist activities against CCR3 and CCR4 (Figure 1A). Among them, Ephedra Herb exhibited the potency to inhibit the cell migration mediated by not only CCR3 and CCR4 but also CCR8 (Figure 1C).

Ephedra Herb, which is listed in the Japanese Pharmacopeia (JPXVI) (The Ministry of Health, Labour and Welfare, 2011) has been used in traditional Chinese formulations and Kampo, formulations for inducing perspiration, healing a cold, relieving cough, and dilating the bronchial tubes. We showed that maoto, a Kampo formulation containing Ephedra Herb, inhibits the cell migration mediated by CCR3, CCR4, and CCR8 (Figure 4). In addition to Ephedra Herb, maoto consists the following three more medical herbs: Glycyrrhiza, Apricot Kernel, and Cinnamon Bark. However, the extracts of Glycyrrhiza, Apricot Kernel, and Cinnamon Bark exhibited no inhibitory effects on CCR3 and CCR4 (Table 1 and Figure 1A). Therefore, we concluded that Ephedra Herb plays a central role in the inhibition of CCR3 and CCR4 (Figures 2, 3). In turn, Ephedra Herb and Ephedra Herb-containing Kampo formulations such as maoto would effectively suppress T_H_2-mediated allergic inflammation. Historically, maoto has been widely used for the treatment of febrile symptoms caused by viral infection. In addition it has recently been shown to suppress ovalbumin-induced asthma and inhibit the recruitment of eosinophils into lung tissues in mice (Ma et al., 2014). Furthermore, it has been shown to decrease IL-4 levels and increase IFN-γ levels in the bronchoalveolar lavage fluid (Ma et al., 2014). In this regard, T_H_2 cell-derived cytokines such as IL-4, IL-5, and IL-13 play a critical role in the pathogenesis of allergic reaction (Brandt and Sivaprasad, 2011); IL-4 and IL-13 stimulate IgE production, and IL-5 is responsible for eosinophil growth, differentiation, migration, activation, and survival (Del Prete, 1998). Based on these findings, Ephedra Herb-containing Kampo formulations such as maoto appear to possess potent activities to strongly affect the T_H_1/T_H_2 balance.

Maoto is prescribed to patients with influenza in Japan. A recent clinical study has demonstrated that maoto has equivalent clinical efficacy to that of neuraminidase inhibitors such as oseltamivir and zanamivir (Nabeshima et al., 2012). Ephedra Herb inhibits the growth of influenza virus A/PR/8/34 (H1N1) by suppressing acidification of cellular components such as endosomes and lysosomes that are essential for the uncoating process of influenza virus in host cells (Mantani et al., 1999). These observations suggest that maoto may also have direct anti-influenza virus activities. T_H_1 cells mainly produce IFN-γ, IL-2, and IL-12 and play a critical role in cell-mediated immune responses and therefore the clearance of viral infection (Lucin et al., 1992; Schijns et al., 1995); T_H_1 polarization is essential for the protective activity against influenza (Gu et al., 2011). In this study, we demonstrated that maoto selectively inhibits chemotaxis mediated by the T_H_2-relevant chemokine receptors (Figure 4). Taken together, one of the underlying mechanisms by which maoto exhibits the anti-influenza activities might be to suppress T_H_2 cell-mediated immune responses by inhibiting cell migration of T_H_2 cells toward inflammation sites.

As described above, Ephedra Herb contains an alkaloid component ephedrine, which has bronchodilating activities as wells as anti-inflammatory effects. Nevertheless, ephedrine exhibited no inhibitory effect on the chemotaxis mediated by CCR3, CCR4, and CCR8 in this study. In contrast, we demonstrated that the EtOAc-insoluble fraction (fraction 2) and the CH_3_Cl-soluble fraction (fraction 3) of Ehpedra Herb include antagonistic components against CCR3, CCR4, and CCR8 (Figures 4A,B). Further investigation of compounds responsible for the antagonist activities in these fractions may lead to develop novel therapeutic agents for allergic diseases.

Among the tested crude drugs/herbs except for Ephedra Herb, Cornus Fruit inhibited the CCR3-mediated chemotaxis, and Rhubarb inhibited the CCR4-mediated chemotaxis, respectively. Cornus Fruit, which is listed in JPXVI, has been used for improving liver and kidney functions in Kampo medicine. Rhubarb, which is also listed in JPXVI, has been used as laxative and anti-inflammatory agents. These crude drugs/herbs also may have potent anti-allergic activities. Further examinations are indispensable to clarify the detail mechanisms of these components and identify responsible constituents.

In the current study, we successfully identified three crude drugs/herbs with the antagonist activity against CCR3 and CCR4 by chemotaxis assays from 80 crude drugs/herbs. In particular, we demonstrated that Ephedra Herb is a potential medical agent for T_H_2-mediated allergic diseases by inhibiting the cell migration mediated by the T_H_2-relevant chemokine receptors such as CCR3, CCR4, and CCR8. This approach using a large crude drug/herb library relevant to Kampo formulations and chemotaxis assays appears to increase the opportunity to identify compounds with immune regulations.

## Author contributions

KM, MF, and TN prepared the manuscript. KM and TN performed the immunological examinations. KK, TM, and MJ handled the crude drug/herb library, which was supervised by NS and IS. OY and TN supervised the entire study.

### Conflict of interest statement

The authors declare that the research was conducted in the absence of any commercial or financial relationships that could be construed as a potential conflict of interest.
